# Analysis of CD83 antigen expression in human breast fibroadenoma and adjacent tissue

**DOI:** 10.1590/S1516-31802011000600006

**Published:** 2011-12-01

**Authors:** Marcus Nascimento Borges, Gil Facina, Ismael Dale Cotrin Guerreiro Silva, Angela Flávia Logullo Waitzberg, Afonso Celso Pinto Nazario

**Affiliations:** I MD, Gynecologist and Mastologist, Department of Gynecology, Universidade Federal de São Paulo, Escola Paulista de Medicina (Unifesp-EPM), São Paulo, Brazil.; II MD, PhD. Adjunct Professor, Department of Gynecology, Universidade Federal de São Paulo, Escola Paulista de Medicina (Unifesp-EPM), São Paulo, Brazil.; III MD, PhD. Associate Professor, Department of Gynecology, Universidade Federal de São Paulo, Escola Paulista de Medicina (Unifesp-EPM), São Paulo, Brazil.; IV MD. PhD. Adjunct Professor, Department of Pathology, Universidade Federal de São Paulo, Escola Paulista de Medicina (Unifesp-EPM), São Paulo, Brazil.; V MD, PhD. Full Professor, Department of Gynecology, Universidade Federal de São Paulo — Escola Paulista de Medicina (Unifesp-EPM), São Paulo, Brazil.

**Keywords:** Fibroadenoma, Antigen-antibody complex, Antigen-presenting cells, Major histocompatibility complex, Immunohistochemistry, Fibroadenoma, Complexo antígeno-anticorpo, Células apresentadoras de antígenos, Complexo principal de histocompatibilidade, Imunoistoquímica

## Abstract

**CONTEXT AND OBJECTIVE:**

Dendritic cell maturation is considered essential for starting an immune response. The CD83 antigen is an important marker of dendritic cell maturation. The objectives here were to analyze CD83 antigen expression in human breast fibroadenoma and breast tissue adjacent to the lesion and to identify clinical factors that might influence this expression.

**DESIGN AND SETTING:**

This was a retrospective study at a public university hospital, in which 29 histopathological samples of breast fibroadenoma and adjacent breast tissue, from 28 women of reproductive age, were analyzed.

**METHODS:**

The immunohistochemistry method was used to analyze the cell expression of the antigen. The antigen expression in the cells was evaluated by means of random manual counting using an optical microscope.

**RESULTS:**

Positive expression of the CD83 antigen in the epithelial cells of the fibroadenoma (365.52; standard deviation ± 133.13) in relation to the adjacent breast tissue cells (189.59; standard deviation ± 140.75) was statistically larger (P < 0.001). Several clinical features were analyzed, but only parity was shown to influence CD83 antigen expression in the adjacent breast tissue, such that positive expression was more evident in nulliparous women (P = 0.042).

**CONCLUSIONS:**

The expression of the CD83 antigen in the fibroadenoma was positive and greater than in the adjacent breast tissue. Positive expression of the antigen in the adjacent breast tissue was influenced by parity, and was significantly more evident in nulliparous women.

## RESUMO

**CONTEXTO E OBJETIVOS:**

A maturação da célula dendrítica é considerada essencial para o início da resposta imune. O antígeno CD83 é um importante marcador da maturação da célula dendrítica. Os objetivos são analisar a expressão do antígeno CD83 no fibroadenoma mamário humano e no tecido mamário adjacente à lesão e identificar fatores clínicos que possam influenciar esta expressão.

**TIPO DE ESTUDO E LOCAL:**

Este é um estudo retrospectivo, realizado em um hospital público universitário, onde 29 amostras histopatológicas de fibroadenomas de mamas e de tecidos mamários adjacentes, de 28 mulheres em idade reprodutiva, foram analisados.

**MÉTODOS:**

O método de imunoistoquímica foi utilizado na análise da expressão celular do antígeno. A expressão do antígeno nas células foi avaliada por contagem aleatória e manual utilizando-se microcópio de luz.

**RESULTADOS:**

A expressão positiva do antígeno CD83 nas células epiteliais dos fibroadenomas (365,52; desvio padrão ± 133,13) em relação às células do tecido mamário adjacente (189,59; desvio padrão ± 140,75) foi estatisticamente superior (P < 0,001). Vários aspectos clínicos foram analisados, porém, a paridade se mostrou influente na expressão do antígeno CD83 no tecido mamário adjacente, onde a expressão positiva foi mais evidente nas mulheres nulíparas (P = 0,042).

**CONCLUSÕES:**

A expressão do antígeno CD83 foi positiva e mais expressiva no fibroadenoma do que no tecido mamário adjacente. A expressão positiva do antígeno no tecido mamário adjacente foi influenciada pela paridade, sendo significativamente mais evidente nas mulheres nulíparas.

## INTRODUCTION

Fibroadenoma is the most common benign tumor in the human breast, especially in women younger than 35 years old.^1^ Histologically, it presents with both epithelial and conjunctive components, and is therefore considered to be a mixed lesion. The principal characteristic of this lesion is the presence of fibrous stroma with low cellularity that lacks cell atypias.^2^

The main function and most important feature of the immune system is its ability to recognize microorganisms, neoplastic cells and other substances as foreign to the organism. This occurs via identification of antigens that may be recognized as noxious. One of the main processes in this recognition occurs through a specialized protein known as the major histocompatibility complex (MHC); this protein has the capacity to bind peptides and to present them to T cells. There are two main types of MHC proteins, class I and class II, which can present peptides to cytolytic lymphocytes (CD8+) or to auxiliary lymphocytes (CD4+), respectively.^3^

For an immune response to start, antigens have to be captured and presented to the lymphocytes that are specific for that antigen. The cells that have this capacity are denoted antigen-presenting cells (APC), and dendritic cells are the APC type that is most specialized for this function. Dendritic cells capture and retain external antigens to be processed via phagocytosis. In the cytoplasm, peptides of these antigens react and link to MHCs. The dendritic cells then migrate to the secondary lymphoid organs, where the peptide-MHC complexes on the cell membrane are presented to inactive T lymphocytes. These cells recognize the peptide-MHC complex through their surface receptors, thus beginning the immune response.

Dendritic cell maturation is considered essential for the beginning of the immune response.^4^ This maturation is completed after interaction with the T cell and is characterized by loss of endocytic capacity and the expression of accessory molecules that interact with the T lymphocyte receptors and function as costimulators, thereby increasing their adhesion and signaling.^5^ One of these costimulatory molecules is called CD83 and is a marker associated with dendritic cell maturation,^6,7^ CD83 is a member of the immunoglobulin superfamily, and is 45 kDa in size. It is also observed in dendritic cells in peripheral blood.^8^

It is known that abnormal cell behavior can lead to emergence of fibroadenoma presenting with proliferation of conjunctive and epithelial tissues.^9^ The action of the immune system against malignant tumors is evident, but little is known about this process in relation to benign neoplasms. Full understanding of the immune response mechanisms relating to neoplasms may open up new horizons for treating these tumors.

## OBJECTIVES

This study had two objectives: first, to immunohistochemically analyze the expression of CD83 in human breast fibroadenoma cells and adjacent normal breast tissue; and second, to analyze which clinical features could influence the expression of CD83 in these breast tissues.

## METHODS

This was a retrospective study, in which 28 women presenting cases of benign nodules in the breasts were selected. All these women received care from the Benign Diseases Sector of the Discipline of Mastology of the Department of Gynecology of a public university hospital.

The patients were between the ages of 16 and 49 years, and all were of reproductive age. Their clinical, gynecological and colpocytological tests were normal, with the exception of the benign neoplasm in the breast. Patients with endocrine diseases, pregnant women and women who had breastfed within the last twelve months were excluded.^10,11^

This retrospective study involved immunohistochemical analysis on histopathological material that was obtained by means of outpatient surgical procedures between January and December 2006. The patients were placed under local anesthesia, and lumpectomy was performed with a safety margin going into the adjacent breast tissue by at least 1 cm from the lesion, and both types of materials obtained (breast lump and the breast parenchyma tissue adjacent to the lesion) were evaluated histopathologically.^10,12^

The biopsies were separated into two groups initially, according to the type of tissue to be analyzed. The first group (group A) included breast lump material (fibroadenomas), and the second group (group B) included the breast parenchyma tissue adjacent to the lesion. The whole material was prepared in a paraffin block for histopathological analysis. The slides were prepared using a primary CD83 antibody and a secondary antibody (CD83 antibody SEROTEC MCA 1582), and then analyzed.

The slides were prepared using a microtome, by taking slices of thickness 2 mm to 3 mm from the paraffin block. These paraffin slices were mounted on slides that had been prepared with silane-APTS (3-aminopropyltriethoxysilane). The slides were then deparaffinized and hydrated via the following steps: immersion in xylol at 60 °C for 15 minutes; xylol at room temperature for 15 minutes; 100% ethanol (three times) for 30 seconds each; 95% ethanol for 30 seconds; 80% ethanol for 30 seconds; 70% ethanol for 30 seconds; and then washing with distilled water.

The next phase in the preparation was antigen recovery from the paraffin-embedded tissue samples, which is necessary for most of the epitopes investigated.^13^ This was accomplished by microwave irradiation, in which the slides were incubated and buffered with 10 mM citrate (pH 5.0) in a microwave oven at maximum power for two sessions of nine minutes each, with a freezing interval of 20 minutes, followed by washing with distilled water.^14^

Subsequently, the slides were subjected to two 10-minute baths in 3% hydrogen peroxide (H_2_O_2_) in methanol, followed by washing with distilled water and buffering using phosphate-buffered saline (PBS) solution. This was used to block the tissue endogenous peroxidase.

The slides were prepared with specific antibodies using the peroxidase-antiperoxidase (PAP) complex, via the following steps:^15^ the slides were incubated with a specific antibody (diluted in PBS), for 16 to 18 hours at 4 °C in a humid chamber; washed three times in PBS for three to five minutes each; incubated with a specific secondary antibody (diluted in PBS) for 30 minutes at 37 °C in a humid chamber; washed three times with PBS for three to five minutes each; incubated with the PAP complex (diluted in PBS) for 30 minutes at 37 °C in a humid chamber; and washed three times with PBS for three to five minutes each. The antigen binding was then revealed after the slides had been incubated in a substrate of diaminobenzidine (DAB) solution (60 mg%), and it could be observed soon afterwards using an optical microscope. The chestnut-gold precipitate was developed as the final product of the reaction on positive control slides. The slides were then washed with distilled water for three minutes, dyed with Harris's hematoxylin for one minute and washed with distilled water. They were then immersed quickly four times in 0.5% ammonium hydroxide, and washed with distilled water; the slides were dehydrated first in 50% ethanol, followed by 80% ethanol, 95% ethanol, 100% ethanol and finally, three times in xylol. Coverslips were affixed using Entellan, for analysis under an ordinary optical microscope.

As previously discussed, the hormonal actions of estrogen and progesterone can influence cell proliferation in fibroadenomas and breast duct epithelium. Therefore, considering the clinical features presented in this study, the sample was further divided in two groups in relation to the use of hormonal contraceptives: group C consisted of 21 cases of women who were not using hormonal contraceptives, while group D consisted of eight women who were using oral hormonal contraceptives. The analysis of CD83 antigen expression in these cells was accomplished by means of random manual counting using optical microscopy, and the results were categorized according to whether the antigen was expressed as much or not as much in the adjacent breast cells as it was in the fibroadenoma cells. The expression of the antigen CD83 was considered positive in the cells in which chestnut staining in the cytoplasm was identified, independent of the intensity or tonality of the staining, because there is no previous knowledge of analyses on this antibody in these tissues. A thousand cells from each case were analyzed, with five hundred from the fibroadenoma and five hundred from the adjacent breast tissue, for statistical calculation of the antigen expression. Initially, the normal distribution test was done to verify that the data obtained presented normal distribution (group A: P = 0.125 and group B: P = 0.572). Since the data presented normal distribution, we used a parametric test (Student's t test for independent groups) for statistical analysis on the results. The value of P < 0.05 was considered statistically significant.

## RESULTS

Histopathological material from 29 cases of fibroadenomas and adjacent breast tissues were analyzed from 28 women aged between 16 and 49 years, with an average age of 27.2 years. Fifteen were nulliparous (P0), one had only had one pregnancy (P1), six had had two pregnancies (P2), five had had three pregnancies (P3) and one had had five pregnancies (P5). Five women had a family history of breast cancer: three had an aunt with breast cancer; one had a grandmother with breast cancer and another had an aunt and a grandmother with breast cancer. The great majority of the cases (23) did not have family history of breast or ovarian cancers (Table 1). All of the women in the study were of reproductive age, and eight of them were using hormonal contraceptives.

**Table 1:** Clinical factors analyzed in the sample

n	Age (years)	HStatus	BCM	FH	Parity	FP	FA	FAD (cm)	Smoker
1	18	M	No	No	P0	22	1	1.1	No
2	43	M	HT	No	P2	19	1	1.8	Ex
3	25	M	No	No	P1	20	1	2.3	No
4	38	M	LT	Aunt	P3	26	1	1.0	No
5	38	M	LT	No	P3		2	0.9	No
6	23	M	No	No	P0	18	2	1.0	No
7	43	M	CI	Aunt	P2	25	1	2.0	Ex
8	36	M	VC	No	P2		1	1.3	No
9	16	M	No	No	P0		1	3.0	No
10	23	M	No	No	P0	26	1	1.7	No
11	39	M	VC	No	P2	21	2	0.9	No
12	43	M	LT	No	P3	22	1	1.2	No
13	39	M	LT	No	P2	22	1	0.8	No
14	45	M	LT	No	P3	21	1	2.5	No
15	45	M	HT	No	P3		1	1.4	No
16	16	M	No	No	P0	21	3	2.4	No
17	49	M	LT	No	P5	28	1	1.3	No
18	38	M	LT	No	P2		1	0.5	No
19	19	M	No	No	P0		2	1.2	No
20	22	M	No	No	P0		1	1.0	No
21	31	M	OC	No	P0		2	1.5	No
22	32	M	OC	No	P0		3	1.2	No
23	19	M	OC	GMo	P1		7	2.0	No
24	38	M	OC	No	P0		1	2.0	No
25	23	M	OC	Aunt	P0		1	2.0	No
26	25	M	OC	Aunt/GMo	P0		1	2.0	No
27	29	M	OC	No	P0		4	1.7	No
28	17	M	OC	No	P0		1	2.5	No

HStatus = hormonal status; BCM = birth-control method; FH = family history of breast cancer; FP = first pregnancy (age in years); FA = fibroadenoma (number in each case); FAD = fibroadenoma diameter; M = reproductive age; HT = hysterectomy; LT = surgical contraception; CI = coitus interruption; VC = vasectomy; OC = oral contraceptive; relatives: aunt; GMo (grandmother); Ex = former smoker.

Most of the fibroadenomas (19 cases) were solitary, but in five cases, the women had two fibroadenomas, and in two cases the patients had three fibroadenomas each. In another single case there were four lumps, and one other woman presented seven fibroadenomas. In nine of the cases of multiple fibroadenomas, five were bilateral. The largest diameter of the lesions ranged from 0.5 cm to 3 cm (average: 1.52 cm).

Other clinical features, such as age at first childbirth and smoking, were considered. However, they did not present statistical relevance (Table 1).

**Figure 1:**
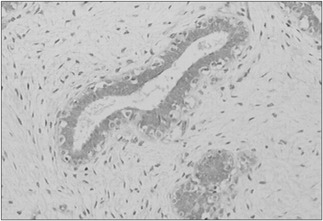
Case 9: Fibroadenoma with positive expression of CD83 antigen (immunohistochemistry staining, optical microscopy - 200 X).

**Figure 2:**
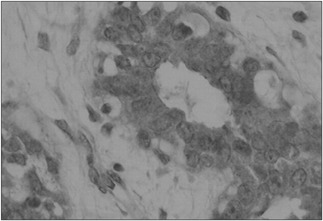
Case 21: Fibroadenoma with positive expression of CD83 antigen (immunohistochemistry staining, optical microscopy - 1000 X).

**Figure 3:**
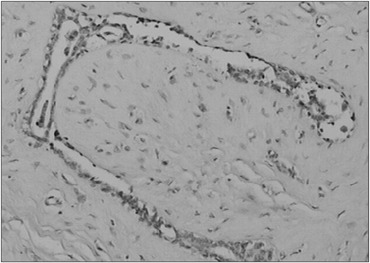
Case 23: Fibroadenoma with negative expression of CD83 antigen (immunohistochemistry staining, optical microscopy - 200 X).

The value of CD83 expression in the fibroadenoma cells and in the adjacent breast cells was analyzed. Positive antigen expression was observed (Figures 1 and 2), on average, in 365.52 (standard deviation [SD] ± 133.13) of the fibroadenoma cells and in 189.59 (SD ± 140.75) of the adjacent breast tissue cells (P < 0.001). In the fibroadenomas, negative antigen expression or non-expression was observed in 134.14 (SD ± 133.40) of the cells (Figure 3), while in the adjacent breast tissue 310.41 (SD ± 134.85) of the cells did not express the CD83 antigen (P < 0.001). From analysis on these results, we observed that the difference in CD83 expression between groups A and B was significant (P < 0.001). Additionally, the negative expression or non-expression of CD83 was also significantly different (P < 0.001). These results confirmed that CD83 expression was much more evident in the fibroadenoma cells (P < 0.001). These values were analyzed in an absolute manner, and they are shown in Table 2.

**Table 2:** Comparison of the CD83 antigen expression in fibroadenoma cells (FA) (group A) and adjacent normal breast tissue (BT) (group B), in absolute numbers; n = 29

Confidence interval statistics					
Variable	Mean/SD	± minimum	maximum	T	P
FA positive	365.52 133.13 (73.10%)	± 314.88 (62.97%)	416.16 (83.23%)		
BT positive	189.59 140.75 (37.92%)	± 138.29 (27.66%)	240.88 (48.18%)	5.662	< 0.001
FA negative	134.14 133.40 (26.83%)	± 83.39 (16.68%)	184.88 (36.98%)		
BT negative	310.41 134.85 (62.08%)	± 259.12 (51.82%)	361.71 (72.34%)	5.671	< 0.001

SD = standard deviation; FA = fibroadenoma; BT = breast tissue; T = Student's t test.

The influence of the use of oral hormonal contraceptives on the cell expression of CD83 was analyzed by using the data obtained from groups C and D. The results from these two groups were analyzed as above.

In group C (i.e. non-users of oral hormonal contraceptives), positive CD83 antigen expression was found on average in 366.38 (SD ± 130.11) of the fibroadenoma cells and in 189.00 (SD ± 137.54) of the adjacent breast tissue cells (P < 0.001). The antigen expression was negative in 133.14 (SD ± 130.49) of the fibroadenoma cells and in 311.00 (SD ± 137.54) of the adjacent breast tissue cells (P < 0.001). These results are presented in Table 3.

**Table 3:** Comparison of CD83 expression in fibroadenoma cells (FA) and adjacent normal breast tissue (BT), in absolute numbers; group C (n = 21) non-users of contraceptives

Confidence interval statistics					
Variable	Mean/SD	± minimum	maximum	T	P
FA positive	366.38 130.11 (73.28%)	± 307.16 (61.43%)	425.60 (85.12%)		
BT positive	189.00 140.75 (37.80%)	± 126.39 (25.28%)	251.61 (50.32)	5.093	< 0.001
FA negative	133.14 133.49 (26.62%)	± 73.74 (14.75%)	192.54 (38.51%)		
BT negative	311.00 137.54 (62.20%)	± 248.39 (49.68%)	373.61 (74.72%)	5.104	< 0.001

SD = standard deviation; FA = fibroadenoma; BT = breast tissue; T = Student's t test.

From analysis on the material from group D (i.e. users of oral hormonal contraceptives), we found an average of 363.25 (SD ± 150.08) cells expressing CD83 in the fibroadenoma, while in the adjacent breast tissue, the average was 191.12 (SD ± 136.71) cells expressing CD83 (P = 0.044). Non-expression was observed in 136.75 (SD ± 150.08) fibroadenoma cells and in 308.87 (SD ± 136.71) cells in the adjacent breast tissue (P = 0.044). These results are presented in Table 4.

**Table 4:** Comparison of CD83 expression in fibroadenoma cells (FA) and adjacent normal breast tissue (BT), in absolute numbers; group D (n = 8), using hormonal contraceptives

Confidence interval statistics					
Variable	Mean/SD	± minimum	maximum	T	P
FA positive	363.25 150.08 (72.65%)	± 237.78	488.72		
BT positive	191.12 136.71 (38.22%)	± 76.83	305.42	2.459	0.044
FA negative	136.75 150.08 (27.35%)	± 11.28	262.22		
BT negative	308.87 136.71 (61.77%)	± 194.58	423.16	2.459	0.044

SD = standard deviation; FA = fibroadenoma; BT = breast tissue; T = Student's t test

The analysis on the results from groups C and D showed that CD83 expression in the fibroadenoma cells was more evident than in the adjacent breast tissue cells, and that in both groups there was a statistically significant difference in the results (respectively P < 0.001 and P = 0.044). In other words, the use of hormonal contraceptives did not appear to have an influence on CD83 expression in this analysis.

Several clinical factors were also considered, such as age, family history of breast cancer, parity, age at first childbirth, diameter of the lump (fibroadenoma), number of fibroadenomas in each patient (multiple or solitary) and smoking (Table 1). The age at first childbirth and smoking appeared to be homogeneous in the samples studied, and therefore lacking in clinical meaning. The other observed features were analyzed statistically, and the results are shown in Table 5. The only feature that presented statistical relevance in relation to CD83 expression was parity and this was only true in relation to the tissue adjacent to the fibroadenoma.

**Table 5:** Analysis of CD83 expression in the fibroadenoma cells (FA) (group A) and in the adjacent breast tissue (BT) (group B), in relation to the clinical features studied (age; family history of breast cancer; parity; number of fibroadenomas in each case; lump diameter)

Variable	FA positive	FA negative	BT positive	BT negative
Age (years)				
≤ 30 years	349.23 ± 135.65	150.00 ± 136.37	216.77 ± 97.99	283.23 ± 97.99
> 30 years	372.53 ± 136.26	127.47 ± 136.26	147.40 ±141.32	352.60 ± 141.32
P	0.655	0.666	0149	0.149
Family history				
Negative	373.17 ± 127.08	126.39 ± 127.41	190.09 ± 131.25	309.91 ± 131.25
Positive	309.00 ± 167.58	191.00 ± 167.58	131.40 ± 93.78	368.60 ± 93.78
P	0.341	0.339	0.335	0.335
Parity				
Nulliparous	391.85 ± 109.37	107.38 ± 109.93	231.08 ± 131.61	268.92 ± 131.61
Others	335.60 ± 150.94	164.40 ± 150.94	135.00 ± 105.63	365.00 ± 105.63
P	0.276	0.27	0.042	0.042
Number of fibroadenomas				
Only 1	344.00 ± 134.30	156.00 ± 134.30	166.21 ± 118.70	333.79 ± 118.70
More than 1	399.11 ± 132.92	99.78 ± 133.56	207.89 ± 143.23	292.11 ± 143.23
P	0.318	0.31	0.424	0.424
Fibroadenoma diameter (cm)				
≤ 1.5	374.53 ± 136.49	124.80 ± 136.97	171.47 ± 151.76	328.53 ± 151.76
> 1.5	346.92 ± 134.91	153.08 ± 134.91	189.00 ± 92.98	311.00 ± 92.98
P	0.596	0.588	0.712	0.712

FA = fibroadenoma; BT = breast tissue.

The CD83 expression in the nulliparous women was positive, on average, in 231.08 (SD ± 131.61) cells and in the other women who had had at least one pregnancy, it was positive in 135.00 (SD ± 105.63) cells in the adjacent breast tissue (P = 0.042). There was no antigen expression in 268.92 (SD ± 131.61) cells in the adjacent breast tissue of the nulliparous women or in 365.00 (SD ± 105.63) cells in the same tissue of the other women (P = 0.042). The analysis on these results in relation to the fibroadenoma cells did not reveal significance (Table 5).

## DISCUSSION

The main objective of this study was to identify CD83 expression in fibroadenoma cells, since the expression of this antigen is an important marker of maturation of the dendritic cell population and, consequently, an indication of immune activity. The immune system has the capacity to react against neoplasms, and the human immune response against tumor cells mainly occurs through activation of cytotoxic T lymphocytes (CD8+). This activation depends on recognition of foreign antigens associated with MHC Class I on the surface of the dendritic cell. Dendritic cells have an antigen-presenting function, and they capture tumor antigens and introduce them to the lymphocyte after migration to the secondary lymph nodes where the immune response begins.^16^ There is a change in the receptor repertoire of the dendritic cell that facilitates its ability to change its location depending on its maturation phase. This increases its effectiveness at finding, capturing and presenting antigens.^17^ Dendritic cells have the capacity to do this in a way that is linked to their maturation, because antigen expression, such as CD83, increases in parallel with the antigen presentation function of dendritic cells.^18^ Lipopolysaccharide (LPS) increases the endocytic activity of dendritic cells and induces CD83 expression. LPS also increases the production of TNF-alpha and E2 prostaglandin (PGE2). Some endotoxins can inhibit these actions of LPS, a phenomenon referred to as tolerance to LPS; this reduces the production of TNF-alpha, interleukin-10 (IL-10) and interleukin-12 (IL-12). In vitro, treatment with TNF-alpha and PGE2 totally rescues CD83 expression and interferon-gamma (IFN-g) release by inducing dendritic cell activity in mixed leukocyte reactions.^19^

Dendritic cell maturation is crucial for the initiation of immune defenses. This process is characterized by a reduction in the capacity to capture antigens and an increase in the capacity to express MHC molecules and costimulators. Maturation occurs when there is an interaction between the dendritic cell and a T cell, followed by a loss of phagocytic ability, and increased strength of interaction with the T lymphocyte receptors; this is due to increased adhesion and signaling capacity with costimulatory molecules.^20,21^ Mature and differentiated dendritic cells that highly express CD83 can be obtained by culturing dendritic cells with granulocyte/macrophage colony-stimulating factor (GM-CSF), interleukin-4 (IL-4) and monocyte-conditioned medium (MCM). These cells have a greater capacity to present antigens and increased mixed leukocyte reaction-stimulatory function.^22^

Identification of a human immune response against neoplasms appears to have an important role in the evolution of neoplastic lesions, and several aspects of the activation of these immune responses and their mechanisms have been elucidated over the last few years. However, many features remain unknown, including the magnitude of the immune response against tumors and the possible clinical applications and effective treatments. The importance of dendritic cell action and activation in the immune system has been implicated in several diseases, for example: viral infection,^23^ parasitic infections such as malaria,^24^ malignant diseases such as colorectal cancer,^25^ Chlamydia infections^26^ and allergic diseases such as atopic eczema and asthma.^27,28^ Many possibilities may open up through studies on the immune system and dendritic cells, thereby making it possible to discover new treatments and vaccines.

There are many reasons why the immune response against tumors may be unsuccessful, and some of these reasons are already known. Every tumor cell expresses MHC class I, but in some situations, this expression is downregulated and the cytolytic T cells are not capable of recognizing them. In tumors with fast growth, there is substantial genetic instability because of the discharge rate of mitoses and loss of the expression of antigens capable of activating the immune system. In such situations, cytolytic T cells cannot be activated because, usually, the tumor cell does not express MHC class II or many other costimulatory molecules that are essential for activation of helper T cells. Many tumor antigens are self-tolerated, and they are thus not recognized as foreign. These immune features and others indicate that it is necessary to maximize the current knowledge about these lesions, so that in the future, treatments that will provide the best treatment of the lesion in a specific manner can be selected.

Because of this, knowledge of CD83 expression in breast fibroadenoma cells becomes very important. The present study shows that there is increased CD83 expression in fibroadenomas, compared with the adjacent breast tissue (P < 0.001), and this could represent more intense immune activity against these benign tumors, probably mediated by antigen-presenting cells (APC). Since there have been no previous evaluations of this antigen in breast tumors, we can propose that CD83 expression is an indicator of the prognosis of lesions and/or is indicative of the future risk of malignant neoplasm. This may even be a good indicator of prognosis for the lesions considered in this study, and suggests that, perhaps in these CD83+ tumors, APC can act with greater effectiveness and, consequently, the immune system can be activated with greater speed and specificity. This knowledge, together with that obtained through future research, may result in new perspectives in relation to effective immunotherapy against this cancer. Positive expression of the antigen CD83 in dendritic cells indicates maturation of these cells and, perhaps, also has the same meaning when expressed in the fibroadenoma epithelial cells, thereby indicating important differentiation between this neoplasia and normal breast tissue.

This study evaluated clinical features that could influence this CD83 expression. The results showed that, of the factors analyzed, the only one that influenced CD83 expression in breast tissue adjacent to the fibroadenoma was the patient's parity. Positive expression of CD83 was more evident in the adjacent breast tissue of the nulliparous women (P = 0.042). There is a coherent association between these data and previous knowledge, which indicates the age at the first childbirth has an influence on breast cancer risk. Having the first childbirth at a younger age reduces the risk of breast cancer.^29^ Additionally, large parity can reduce this risk.^30^ The first pregnancy indicates the time at which the physiological maturation of the breast gland is completed, presumably from the intense estrogen, progesterone, HCG and prolactin stimulation. The maturation begins with ductal ramification and growth of the lobule-alveolar structure, until the formation of multiple alveoli is complete and consequent glandular epithelium proliferation occurs. This development is followed by the formation of glandular structures, lobule-alveolar system maturation and secretory epithelium maturation.^31^ These breast tissue modifications, associated with obstetric factors, affect cytological characteristics and the risk of breast cancer, thus indicating that the gland is influenced by pregnancy and may develop the capacity to recognize antigens and activate the immune system.

## CONCLUSIONS

The expression of CD83 in the fibroadenoma cells was positive and greater than in the breast tissue adjacent to the lump (P < 0.001), when analyzed by means of immunohistochemistry. New studies should be conducted in order to define the clinical meaning of this result and investigate the possibility that this knowledge may be important in the pursuit of new immune therapies against breast tumors.

The expression of CD83 in the breast tissue adjacent to the fibroadenoma was influenced by whether the patient was nulliparous or had already had at least one full term pregnancy, and it was significantly greater in nulliparous women (P = 0.042). In this context, the repercussions of pregnancy in breast tissue may affect the immune response; this needs to be better characterized, given that CD83 expression in breast tissue adjacent to the fibroadenoma was influenced by the patient's history of pregnancy (P = 0.042).
